# Developing and Applying the Propensity Score to Make Causal Inferences: Variable Selection and Stratification

**DOI:** 10.3389/fpsyg.2017.01413

**Published:** 2017-08-17

**Authors:** Jill L. Adelson, D. B. McCoach, H. J. Rogers, Jonathan A. Adelson, Timothy M. Sauer

**Affiliations:** ^1^Department of Counseling and Human Development, University of Louisville, Louisville KY, United States; ^2^Department of Educational Psychology, University of Connecticut, Storrs CT, United States; ^3^Adelson & Co., Louisville KY, United States; ^4^LEAP, Louisville KY, United States

**Keywords:** propensity score, stratification, Monte Carlo simulation, effect size, variable selection

## Abstract

This Monte Carlo simulation examined the effects of variable selection (combinations of confounders with four patterns of relationships to outcome and assignment to treatment) and number of strata (5, 10, or 20) in propensity score analyses. The focus was on how the variations affected the average effect size compared to quasi-assignment without adjustment for bias. Results indicate that if a propensity score model does not include variables strongly related to both outcome and assignment, not only will bias not decrease, but it may possibly increase. Furthermore, models that include a variable highly related to assignment to treatment but do not also include a variable highly related to the outcome could increase bias. In regards to the number of strata, results varied depending on the propensity score model and sample size. In 75% of the models that resulted in a significant reduction in bias, quintiles outperformed the other stratification schemes. In fact, the richer that the propensity score model was (i.e., including multiple covariates of varying relationships to the outcome and to assignment to treatment), the more likely that the model required fewer strata to balance the covariates. In models without that same richness, additional strata were necessary. Finally, the study suggests that when developing a rich propensity score model with stratification, it is crucial to examine the strata for overlap.

## Introduction

Although randomized studies may be the ideal, the nature of the subjects and settings as well as economic constraints and ethics prohibit many large-scale experimental studies in social science or psychological research. When data are observational or quasi-experimental, the subjects who receive a particular intervention (the treatment group) and those who do not (the comparison group) may have systematic pretreatment differences that are related to the outcome of interest, such as different ability levels (Rosenbaum and Rubin, 1983; Joffe and Rosenbaum, 1999; D’Agostino and Rubin, 2000; Braitman and Rosenbaum, 2002). Differences in outcomes between the two groups may reflect these differences, which are regarded as bias, rather than effects of the intervention, whether it is a program, policy, or other “treatment.” Therefore, when examining treatment effects in observational studies to make causal inferences, psychologists must make analytical adjustments for the overt bias, that is, the measured pretreatment differences [Note that even after adjusting for overt bias, researchers cannot assume that treatment effects are measured without bias unless all necessary covariates are observed and measured without error (Rosenbaum and Rubin, 1983)].

The first step in propensity-score methods is to create the propensity score. One of the goals of this study is to examine whether researchers should focus only on variables related to assignment (as historically was done) or also to covariates related to the otucome. Once the propensity score is created, the author must choose a propensity-score method. Frequently used propensity-score methods include stratification, covariate adjustment, and matching (for more detailed description, see Austin, 2007, 2008; Austin et al., 2007; D’Agostino, 2007; Lunt et al., 2009; Shadish and Steiner, 2010). Of particular interest to this study is the method of stratifying subjects on a propensity score (Rosenbaum and Rubin, 1983) to estimate treatment effects. In this method, the researcher creates balanced strata based on the propensity score, and the researcher must make a decision of how many strata to create. Some examples of propensity score stratification in educational psychology research include studies by Hong and Raudenbush (2005), Adelson et al. (2012), Dağli and Jones (2013), and Goos et al. (2013). Despite the widespread usage of propensity-score methods, limited research has been conducted on variable selection for the propensity score model or on the number of strata needed to minimize bias in the estimate of the treatment effect. The purpose of this Monte Carlo simulation study was to examine the effects of variable selection and number of strata in propensity score analyses.

## Literature Review

### The Propensity Score

The propensity score represents the conditional probability of membership in the treatment group based on measured pretreatment background variables (Rosenbaum and Rubin, 1984, 1985; Joffe and Rosenbaum, 1999). Stated formally, the propensity score *e*(*x*) is the conditional probability of belonging to the treatment group given the observed pretreatment variables, *x*, denoted as

$$\begin{array}{c} e\text{​}\left(x\right)=pr\text{​}\left(z=1|x\right)\text{​}, \end{array}$$

where *z* = 1 for subjects in the treatment group and *z* = 0 for comparison subjects (Rosenbaum and Rubin, 1983). By making analytical adjustments based on the relationship between pretreatment characteristics and treatment group membership, researchers attempt to reconstruct a situation similar to random assignment after the fact, although this is limited to observed pretreatment characteristics only and does not adjust for hidden bias (Braitman and Rosenbaum, 2002). By adjusting for these pretreatment confounders through the propensity score, researchers obtain “quasi-randomization of treatment groups to minimize bias and to better estimate the true effects of treatment” (Newgard et al., 2004, p. 954).

The propensity score creates balance between treatment and comparison groups in the sense that when the propensity score is held constant – that is, when subjects have the same propensity score – the joint distribution of the pretreatment covariates is the same across the two groups. Because the propensity score adjusts for this vector of covariates, the researcher assumes strongly ignorable treatment assignment given those observed covariates, although that assumption only holds to the degree that all relevant pretreatment covariates are measured (Rosenbaum and Rubin, 1983). Thus, by using the propensity score, researchers can compare subjects with equal probability of being in the treatment group based on the covariates but who are, in fact, in different groups (Rosenbaum and Rubin, 1983, 1984; Shadish et al., 2006).

Researchers have several options regarding how to use the propensity score in their analyses, including matching, weighting (Thoemmes and Ong, 2016) and stratification (also called subclassification). One method is stratification, or grouping subjects based on their propensity score (either with equal-interval strata in which the range of propensity scores is divided by the number of strata so that each stratum is equal width or with equal-size strata in which the strata are divided according to percentiles so that each stratum includes the same percent of participants, with varying ranges of propensity scores in each stratum). Once researchers stratify the subjects on the propensity score, they can compare the effects of treatment between the two groups within the same strata, or subclass, thus controlling for overt bias (Cochran, 1965, 1968; Rosenbaum and Rubin, 1984). Stratification has several advantages, including that it is easy to implement and to interpret, it often is convincing to non-technical audiences, and it easily can accommodate additional model-based adjustments (Rosenbaum and Rubin, 1983, 1984; Rosenbaum, 1987). According to Rosenbaum and Rubin (1983), propensity score stratification is easier and almost as efficient as matching and also is less sensitive to non-linearities in the relationship between propensity scores and outcomes than other methods. Furthermore, researchers can examine treatment effects within particular strata, such as the stratum for which treatment is most likely to be effective.

Because the propensity score is a scalar function of pretreatment covariates that “summarizes the information required to balance the distribution of the covariates” (Rosenbaum and Rubin, 1984, p. 516), researchers can use the propensity score alone to form strata that may balance all covariates, or make the conditional distribution of the covariates given the propensity score equal for those in the treatment and those in the comparison conditions, regardless of the number of observed covariates from which it was constructed (Rosenbaum and Rubin, 1983). This makes stratification much more feasible than stratifying on individual covariates, which can become extremely unwieldy, as even with dichotomous covariates, for *p* covariates there are 2^p^ subclasses (Cochran, 1968). Note that covariate balance is an empirical issue that must be checked, as the distributional balance of the covariates is expected rather than guaranteed (similar to in randomization). Two methods to check for balance include (a) regressing each covariate and the logit of the propensity score on the treatment assignment, controlling for *S*-1 dummy indicators for the *S* propensity strata and their interactions with treatment assignment, and (b) conducting 2 × *S* (Conditions × Strata) ANOVAs, using both the propensity score and each predictor individually as dependent variables. Rubin (2001) presents three criteria for assessing the adequacy of the regression adjustment: (a) the difference in the means of the propensity scores in the groups being compared must be small, (b) the ratio of the variances of the propensity score in the treatment and comparison groups must be close to one, and (c) the ratio of the variances of the residuals of the covariates after adjusting for the propensity scores must be close to one.

Stratification on the propensity score does have some limitations. As mentioned previously, although this method balances the distribution of the observed pretreatment covariates included in the propensity score (overt bias), it cannot balance unobserved covariates (hidden bias) except to the extent that they are correlated with observed covariates (Rosenbaum and Rubin, 1984). Additionally, in practice, strata generally will not be exactly homogenous in the propensity score that was used to create the strata. As a result, the estimate of the effect may contain some residual bias due to measured pretreatment covariates (Rosenbaum and Rubin, 1983).

Rosenbaum and Rubin (1983, 1984) have shown that five equal-size propensity score strata (quintiles) can remove over 90% of the bias due to each of the pretreatment covariates used to construct the propensity score (depending on the variables that are included, as strong ignorability must hold), just as Cochran (1968) indicated that stratifying on individual covariates would do. That being said, the literature on propensity score stratification provides several different recommendations for the number of strata. Rosenbaum and Rubin (1983, 1984), Rosenbaum (2002a), and Shadish et al. (2006) recommend stratifying on quintiles. On the other hand, Imai and van Dyk (2004) recommend that researchers examine the sensitivity of their results by repeating the analysis with different stratification schemes as “results may be sensitive to the number of subclasses” (p. 857). This recommendation is supported by Hullsiek and Louis (2002), who indicate that with a large data set (such as those typically used with propensity score analysis), researchers might need to form more than five strata to achieve maximal bias reduction [Alternatively, researchers can address residual bias after stratification by running regression adjustments within each stratum (Rubin, 2001)]. Researchers using propensity score stratification have differed in which approach they have used. For instance, Leow et al. (2004) stratified high school students into quintiles based on a propensity score. On the other hand, Hong and Raudenbush (2005) divided schools into seven strata, dropping the lowest propensity stratum because the comparison group did not have matches in the treatment group, and divided students into 14 propensity score strata, dropping the last stratum as 8 students in the treatment group had no matches in the comparison group. They made their decision for seven and 14 strata based on balance indices.

### Selecting Variables to Construct Propensity Scores

Parsimony is not essential when estimating propensity scores because the propensity score operates as a scalar function and summarizes the collection of pretreatment covariates when estimating the treatment effect. To minimize bias, the goal of propensity score analysis is to balance treatment and comparison subjects on as many pretreatment covariates as possible. Omitting even pretreatment variables that are weakly predictive of the outcome will have biasing effects that may override the statistical efficiency of not including them (Rubin, 1997; Newgard et al., 2004). In fact, Rubin and Thomas (1996) recommended that researchers retain non-statistically significant predictors. If researchers do construct the propensity score from only pretreatment covariates that are statistically significantly different between the treatment and comparison groups, they are failing to take into account the relationship between the covariates and the outcome, are relying heavily on sample size and not practical relevance, and are considering the covariates in isolation rather than collectively (Rosenbaum, 2002b). Furthermore, if iterative model-building algorithms such as stepwise regression are used to predict assignment, the researcher may miss important confounders that while only weakly related to assignment are strongly related to the outcome.

Researchers have conducted limited studies on the effects of using variables with varying relationships to the outcome and to assignment to treatment. Brookhart et al. (2006) explored the effects of using variables with varying relationships to the outcome and to assignment to treatment in two simulation studies. The first study investigated how estimated treatment effects were affected by the inclusion of (a) a variable related both to assignment and to the Poisson-distributed outcome, (b) a variable related to the outcome only, and (c) a variable related to assignment only, independently and in all combinations. When they analyzed the data using stratification (based on quintiles), they found that failure to include the variable related to both assignment and outcome yielded a biased estimator. For the second simulation, they included only a single variable that was related to both outcome and assignment, varying the correlation magnitude. The researchers consistently found that the variance of the propensity score estimator may be “slightly more sensitive” to the strength of the relationship between the variable and assignment. Brookhart et al. (2006) found that when they added a single variable with varying magnitudes of correlation to the outcome and assignment, the variance of the estimator decreased proportionally to the sample size. Despite this decrease in variance, the bias due to an omitted confounder remained, regardless of sample size.

The results of Brookhart et al. (2006) simulation studies suggest that when analyzing at least moderate-sized data sets, researchers should not exclude any variable related to assignment from the propensity score model unless they know *a priori* that the variable truly has no relationship to the outcome. Additionally, researchers should include in propensity score models all variables that might relate to the outcome even if they are unrelated to assignment, which also was the recommendation of Rubin and Thomas (1996). In reality, nearly all pretreatment variables will have some relationship to both outcome and assignment, and researchers must adjust for that bias.

## Statement of the Problem

The study reported here built on the work of Brookhart et al. (2006) by further exploring variable selection, except with a normally distributed outcome variable. In this study, we examined the effects of including different combinations of variables with differing relationships to outcome and to assignment, as well as those variables independently. Because the propensity score is “a means of obtaining quasi-randomization of treatment” (Newgard et al., 2004, p. 954), the treatment effects obtained with random assignment were compared to those obtained with a quasi-assignment variable only (a non-random assignment to the treatment condition, correlated with the outcome variable). Then, both of those treatment effects were compared with those obtained with propensity score stratification using models built with the various combinations of covariates. Furthermore, because prior research and theoretical work indicates that the number of strata may affect the estimated treatment effects, this study compared the estimates obtained with varying number of strata.

## Methodology

To explore the variable selection problem and the effects of using different numbers of strata, a Monte Carlo simulation study was performed. A Fortran program written by the authors in Lahey ED for Windows, version 3.80, was used on a Windows platform. To assign individuals to the treatment or comparison group, the program generated an assignment variable that provided for non-random assignment to the treatment condition, as happens in observational or quasi-experimental data. This assignment variable (mean of 0, standard deviation of 1) was specified to have a 0.50 correlation with the outcome, a moderate relationship between assignment and outcome as one might expect in educational settings in which achievement is closely related to programs in which students participate, such as gifted classes or remedial tutoring. Similarly, in many voluntary treatment programs, such as a group for narcotics addiction, participating in the program is closely related to an outcome measure, such as as number of days clean. For individuals with an illness such as HIV, choosing to attend appointments with a doctor or at a clinic is closely related to adherence to a drug regimen. After the assignment variable was created, the subjects were ranked.

To create a dichotomous assignment variable from this normally distributed continuous assignment data that had a correlation of 0.50 with the outcome, the program ranked the subjects according to their continuous assignment variable again assigned the top 50% to the treatment group (1) and the bottom 50% to the comparison group (0). Additionally, to create a dichotomous variable that simulated random assignment (correlation of 0 with the outcome), the program generated a random number for each subject, ranked the subjects according to their random number, and then assigned those in the bottom 50% to the comparison group (0) and those in the top 50% to the treatment group (1).

The program also generated four different types of covariates, each a true confounder but with varying relationships to outcome and assignment. Note that because the propensity score model includes many covariates (thus, being rich in their representation of the potential covariates that relate to the outcome and assignment), these four variables could be considered composite variables made up of many other variables. Variable HH had a high (0.70) relationship with assignment and with outcome, HL had a high (0.70) relationship with assignment but a low (0.20) relationship with outcome, LH had a low (0.20) relationship with assignment but a high (0.70) relationship with outcome, and LL had a low (0.20) relationship with assignment and with outcome. These correlation strengths fall within rules of thumb for a “high positive correlation” and “little if any correlation” (Hinkle et al., 2003, p. 109). From our research, an example of two variables correlated at 0.70 is mathematics achievement scores in spring with reading achievement scores the following winter, and an example of two variables correlated at 0.20 is students’ exceptional motivation to succeed (as rated by their teachers) and their reading achievement at that same time point. All of these covariates had a 0.20 correlation with one another. All variables were created to be normally distributed with a mean of 0 and standard deviation of 1.

One thousand data sets were simulated, each with a sample size of 10,000. Because propensity score analysis is a large-sample method, this is a reasonable sample size. For instance, the Early Childhood Longitudinal Study, Kindergarten Class of 1998-1998 (ECLS-K) had a sample of over 20,000 kindergarteners, High School and Beyond (HSB) had an initial sample of over 28,000 high school students, and the Trends in International Mathematics and Science Study (TIMSS) assessed about 4,000 students *per grade* and *per country*. Moreover, it is only over the long run or in very large samples that randomization can be expected to work “perfectly,” resulting in no difference, or treatment effect, between the two random groups. For example, with a sample size of 10,000 and a true effect size (defined by Cohen’s *d*) of zero, an average effect size for the random model of 0.0001, with a standard deviation of the estimate of 0.0004, was obtained across the 1,000 replications. However, with half that sample size (*n* = 5,000), the average effect size for the random model was 0.011 due to sampling error, and with an even smaller sample size (*n* = 2,500), the average estimate of the effect size was 0.063.

For each simulated data set, logistic regression was used to estimate 15 different propensity scores for each participant, corresponding to all possible combinations of the four covariates (**Table 1**). Then, any treatment participant whose propensity score was outside the range of the comparison group’s propensity scores was eliminated and vice versa, ensuring overlap in propensity scores and that there were appropriate comparison participants for each one remaining in the data set. Although this insures appropriate overlap of participants in the two groups, it does mean that we are no longer able to generalize to the full sample, and that must be noted in an applied study. For each data set and all 15 specifications of propensity score models, across 5, 10, and 20 strata, equal-size strata were created by creating strata at the appropriate percentile (quintiles, deciles, etc.) of the sample distribution of the propensity score. For example, for quintiles, the sample with the lowest 20% of propensity scores were one strata, the sample with the next lowest 20% of propensity scores were another strata, and so on to create five strata, each consisting of 20% of propensity scores. Equal-interval strata originally also were estimated by finding the range of propensity scores and dividing by the number of strata to determine the width of the strata. Overall, equal-interval strata were not as effective as equal-size strata at removing bias and resulted in the retention of fewer viable strata when calculating the average effect size. This is consistent with previous literature, which does not recommend dividing strata into equal-size intervals.

**Table 1:** Propensity score models.

Model	Variables			
1	HH^a^			
2	HL			
3	LH			
4	LL			
5	HH	HL		
6	HH	LH		
7	HH	LL		
8	HL	LH		
9	HL	LL		
10	LH	LL		
11	HH	HL	LH	
12	HH	HL	LL	
13	HH	LH	LL	
14	HL	LH	LL	
15	HH	HL	LH	LL

*^a^The first letter indicates whether the covariate has a high (H) or a low (L) correlation, 0.70 and 0.20, respectively, with the assignment to treatment. The second letter indicates whether the covariate has a high (H) or low (L) correlation, 0.70 and 0.20, respectively, with the outcome.*

Propensity score models typically are used to determine the effect of a treatment. Rather than focusing on statistical significance of the differences between treatment and comparison groups (the estimand), the primary interest of this study was the average effect size of the treatment for each model over the 1,000 replications. That is, our model was examining the difference in average outcome scores for the treatment and comparison groups across the strata. As a measure of effect size, Cohen’s *d*, the difference between the comparison and treatment means divided by the pooled standard deviation for those means, was used. The average quasi-experimental and randomized effect size for each data set were estimated by finding the effect size within strata and averaging over strata. Also, the 95% confidence interval around that estimate was estimated by adding/subtracting 1.96 times the standard deviation of the effect size to/from the average effect size. After performing all 1,000 replications, the overall average effect size (*ModelAverageEffect*) for each of the 15 models for each specified number of strata was found. Then, those model average effect sizes were used to determine the proportion reduction in bias, which was calculated with the following:

$$\begin{array}{c} Proportion\;reduction\;in\;bias=\frac{Quasi\;Average\;Effect-\left|Model\;Average\;Effect\right|}{Quasi\;Average\;Effect}, \end{array}$$

where the *QuasiAverageEffect* was equal to the average effect size for a model in which the quasi-assignment variable was used without accounting for any of the pretreatment covariates. The absolute value of the average effect size for the model was used to account for the fact that some models resulted in a negative estimate of the effect size, which also indicates bias.

In summary, this simulation study compared the performance of the 15 various specificiations of propensity score models with 5, 10, and 20 equal-size strata. To have a randomized effect that approximated 0, a sample size of 10,000 was used. Because propensity score analysis is a large-sample technique, the focus was on how the different propensity score models and number of strata affected the effect size (Cohen’s *d*) rather than the rejection rate of the null hypothesis, which is strongly dependent on sample size. To evaluate the performance of the various propensity score models, the simulated results were used to determine the average effect size when participants were randomly assigned, the average effect size when participants were assigned based on quasi-assignment without adjustment for the covariates, the average effect size across the strata for each model, and the proportion reduction in bias.

## Results

### Comparison Models

With a sample size of 10,000, the average effect size for the random model was 0.0001, and the standard deviation of the estimate was 0.0004, making the 95% confidence interval (-0.0007, 0.0009). However, for the model with participants grouped by the quasi-assignment variable without adjustment for the covariates, the average effect size was 0.893, with a standard deviation of 0.001, making the 95% confidence interval (0.891, 0.895). This estimate represents the bias that the researcher incurs due to using quasi-experimental, or observational, data in which assignment is not random but is related to the outcome measure. Despite the “treatment” not having an effect, a large average effect size due to the non-random assignment was observed.

### Propensity Score Models

**FIGURE 1:**
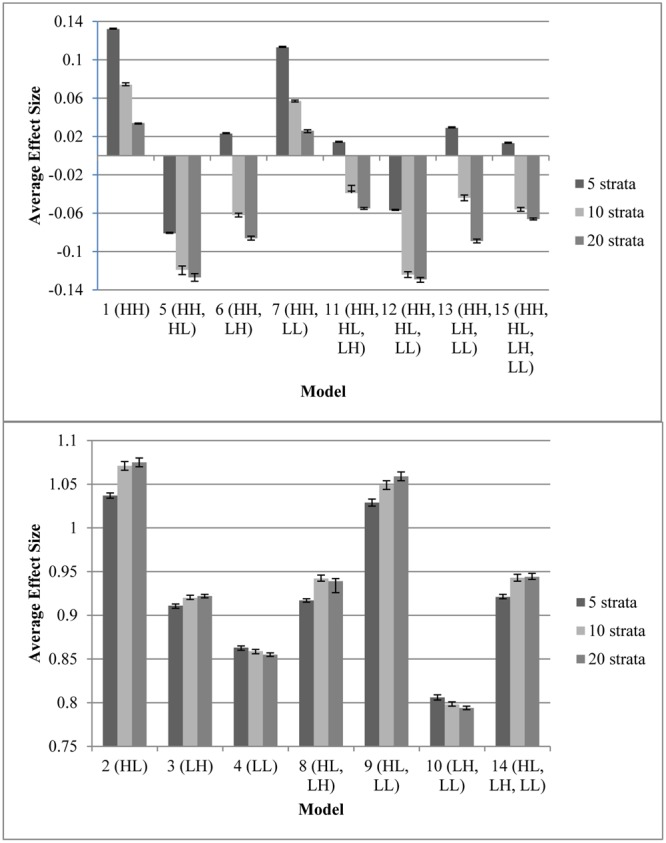
Average effect sizes from the propensity score models with varying number of specified equal-size strata, with 95% confidence interval for the estimate. Any effect size present is due to bias because the model on which these data were based had an effect size of 0. **(Top)** Models with minimal bias. **(Bottom)** Models with extensive bias. Note that participants with a propensity score outside the range of the other group were eliminated prior to stratification; therefore, sample sizes varied as different models resulted in differing overlap in treatment and control propensity scores **(Table 2)**.

**FIGURE 2:**
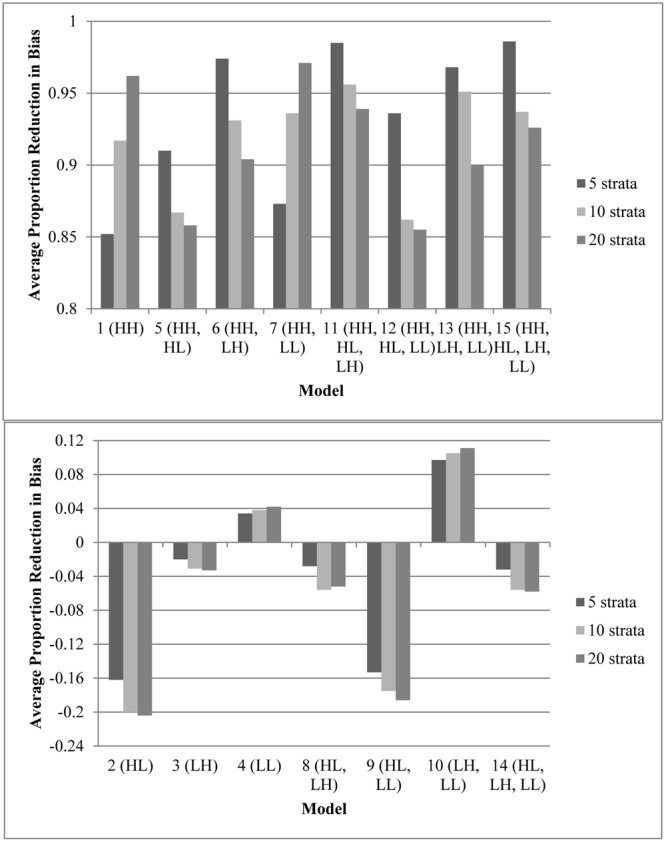
Average proportion reduction in bias for the propensity score models with varying number of specified equal-size strata. The model with no adjustments for the covariates had an effect size of 0.893. This figure indicates the proportion of reduction in that bias. **(Top)** Models that reduced bias by 85% or more. **(Bottom)** Models that removed less than 15% of bias (or increased biase). Note that participants with a propensity score outside the range of the other group were eliminated prior to stratification; therefore, sample sizes varied as different models resulted in differing overlap in treatment and control propensity scores **(Table 2)**.

**Figure 1** displays the average effect sizes observed from the 15 propensity score models with 5, 10, and 20 equal-size strata created at the appropriate percentiles of the sample distribution and the 95% confidence intervals of those estimates. Effect size estimates closer to zero (those for models that contain HH, the variable with a high correlation to both assignment and outcome) show better adjustment for bias due to non-random assignment and better approximation to the effect size obtained with random assignment. On the other hand, effect size estimates above 0.893, the average effect size for the quasi-assignment model, show increased rather than decreased bias. **Figure 2** shows the proportion reduction in bias for the 15 propensity score models with the various numbers of strata. When interpreting the results, one must note that non-overlapping propensity scores between the treatment and comparison groups were eliminated, resulting in reduced sample sizes for some models (see **Table 2** for the average sample size for each model). Although the majority of models included more than 95% of the treatment sample, comparison sample, and total sample, four models only retained about 80% of the total sample: Model 1 (HH), Model 7 (HH, LL), Model 11 (HH, HL, LH), and Model 13 (HH, LH, LL). In addition to reduced sample sizes, when this occurs, the researcher must be careful when interpreting the results to identify who was not included in the analyses and acknowledge to whom the results generalize. The type of participant that was in one group and not the other may be of substantive interest, such as if one group included students with higher or lower achievement, motivation, intelligence, parental involvement, socio-economic status, etc., than were found in the other group.

**Table 2:** Average sample size^a^ for the propensity score models.

Model	Treatment	Comparison	Total
1 (HH)	4859 (97%)	4834 (97%)	9693 (97%)
5 (HH, HL)	3776 (76%)	4191 (84%)	7967 (80%)
6 (HH, LH)	4876 (98%)	4826 (97%)	9702 (97%)
7 (HH, LL)	4849 (97%)	4805 (96%)	9654 (97%)
11 (HH, HL, LH)	3793 (76%)	4200 (84%)	7993 (80%)
12 (HH, HL, LL)	3778 (76%)	4266 (85%)	8044 (80%)
13 (HH, LH, LL)	4849 (97%)	4833 (97%)	9682 (97%)
15 (HH, HL, LH, LL)	3797 (76%)	4265 (85%)	8062 (81%)
2 (HL)	4837 (97%)	4855 (97%)	9692 (97%)
3 (LH)	4996 (99+%)	5000 (100%)	9996 (99+%)
4 (LL)	4998 (99+%)	4998 (99+%)	9996 (99%)
8 (HL, LH)	4829 (97%)	4795 (96%)	9624 (96%)
9 (HL, LL)	4861 (97%)	4857 (97%)	9718 (97%)
10 (LH, LL)	4999 (99+%)	4998 (99+%)	9997 (99+%)
14 (HL, LH, LL)	4851 (97%)	4830 (97%)	9681 (97%)
Overall	4597 (92%)	4703 (94%)	9300 (92%)

*^a^Participants with a propensity score outside the range of the other group were eliminated prior to stratification. Therefore, sample sizes varied as different models resulted in differing overlap in treatment and comparison group propensity scores. The percent of the original 5000 treatment, 5000 comparison, and 10,000 total participants is given in parentheses.*

Every model that removed at least 90% of the bias (average effect size of ≤ |0.089|, which is 90% of the average effect size when participants were grouped based on quasi-assignment without adjustment for the covariates; i.e., 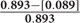 = **0.90**) contained HH (Models 1, 5, 6, 7, 11, 12, 13, and 15). All models that omitted HH removed less than 15% of the bias, and in some cases, increased the amount of bias. In fact, without this variable, the proportion of reduction in bias was near zero and sometimes even negative. Interestingly, for any model containing HH and at least one other variable with a strong correlation to assignment or outcome, quintiles were the most effective at removing bias, whereas in models that only included HH or HH along with LL, quintiles reduced the bias by less than 90% but 20 strata performed extremely well. In fact, those two models were the best performing when 20 strata were used. On the other hand, with quintiles, the models behaved in a manner more similar to that expected, with the model with all four covariates and the model with all covariates except LL reducing the bias most. Following those models, the next best models with quintiles included the covariate strongly related to the outcome but not to assignment to treatment (LH). Looking at the top four models at reducing bias, one sees that the two models that show up in all three stratification schemes are the models that included HH, HL, and LH (Models 11 and 15). For all stratification schemes, the worst two models (Models 2 and 9) did not include any variables with a strong relationship to outcome but did include a variable with a strong relationship to assignment. In fact, any time HL was included with HH, the amount of bias was increased rather than reduced.

In terms of overlap of participants in the treatment and comparison groups, the more predictors that were strongly related to assignment to treatment, the less the overlap. Any model containing both HH and HL (Models 5, 11, 12, and 15) had an overlapping sample size of about 8,000, with more people in the comparison group than the treatment group, whereas all other models retained greater than 95% of the sample. This highlights the fact that when the assignment to treatment is not random but even moderately related to the outcome, there are great differences between the treatment and comparison groups. With 10 or 20 strata, Models 5 (HH, HL) and 12 (HH, HL, LL) were the only two models that contained HH and failed to reduce the bias by at least 90%. This result may be due to the lack of overlap of propensity scores between treatment and comparison groups in these models, which reduced the sample size to around 8,000. In general, the models with smaller samples performed better with quintiles, perhaps because with the reduced sample size, the number of subjects in each stratum was reduced. Thus, when the data became sparser as the number of strata increased.

## Conclusion

Perhaps most importantly, this simulation study revealed that if a propensity score model does not include variables strongly related to *both* outcome and assignment, bias will not decrease and may possibly increase when using the stratification method. Although also including variables with weak associations either to outcome, to assignment, or to both tends to result in a greater decrease in bias (particularly with quintiles and deciles), without a variable (or composite of variables) strongly related to both outcome and assignment, the propensity score is ineffective at removing bias from the estimated effect size. Thus, researchers must be sure to include variables like a pretest score, which would have a high correlation to the assignment to or inclusion in many “treatments” as well as to an outcome like posttest score. Additionally, including a variable highly related to assignment but not also including a variable highly related to outcome could, in fact, be detrimental, causing an increase in bias, which supports the findings of Brookhart et al. (2006). In fact, our study provides further evidence of the importance of the variable highly related to the outcome because it found that including only a variable with a low correlation to both assignment and outcome was less detrimental than including a variable with a strong relationship with assignment but not including a variable with a strong relationship with outcome.

Overall, the findings are consistent with the advice of Rubin and Thomas (1996), Rubin (1997), Newgard et al. (2004), and Brookhart et al. (2006) that researchers should include in propensity score models all variables thought to be related to the outcome. Because all variables considered in this simulation had at least a weak relationship with both outcome and assignment, they are empirical confounders for the study. According to Brookhart et al. (2006), “including such a covariate in a [propensity score] model removes the non-systematic bias due to the chance association between the covariate and [assignment]” (p. 1155). Removing this non-systematic bias brought the estimated effect size closer to the true effect size of zero. This finding has important implications for researchers as they plan their study and as they select variables from pre-existing data sets.

In terms of number of strata, this study highlights Imai and van Dyk (2004) comment that researchers must be aware that their results may vary depending on the number of strata. Depending on the covariates and the sample size, 5, 10, and 20 strata performed differently, although in six of the eight models that resulted in a significant reduction in bias, quintiles outperformed the other stratification schemes. With quintiles, as long as two sets of covariates with a strong relationship to the outcome were included, more than 95% of the bias was removed, and when at least one variable with a strong relationship with either outcome or assignment was included with the HH variable, at least 90% of bias was removed. These results support the recommendations of Rosenbaum and Rubin (1983, 1984), Rosenbaum (2002a), and Shadish et al. (2006) that when the assumption of strongly ignorable assignment to treatment is met, quintiles may remove 90% of the bias. Based on the results of this study, the richer the propensity score model in terms of varying relationships to outcome and assignment to treatment, the more likely that fewer the strata will be needed to balance the covariates. However, without that same richness, additional strata may be necessary.

Finally, this simulation study should serve as a cautionary tale to applied researchers using propensity score analysis. When developing a rich propensity score model and using stratification, it is crucial to examine the strata for overlap. With models that included more than one covariate with a strong relationship to assignment to treatment, the overlapping sample size was only about 80% of the original sample size. However, models that did not include the variable strongly related to assignment but not to outcome (HL) still removed at least 90% of the bias for all stratification schemes as long as HH also was included in the model, and for those models, more than 95% of the sample had overlapping propensity scores.

### Limitations

The results from this simulation are consistent with theoretical results (e.g., Rubin and Thomas, 1996) as well as previous simulation results (e.g., Brookhart et al., 2006). However, the amount of bias reduced is dependent on the generated sample data and the specification of the data-generating process. For instance, only certain correlations between the covariates and assignment, outcome, and other covariates were considered. Although different numbers of strata were investigated, this study did not explore the effects of using other propensity score methods, such as different methods of matching, or the effects of using an outcome model with additional covariates or a hierarchical linear outcome model. Finally, because of the “gold standard” to which the results were compared in this study, we used a large sample size of 10,000. However, many applied studies include fewer observations. These results may not be generalizable to studies with small sample sizes.

Although researchers should strive for a rich propensity score model with many covariates, only one to four covariates were included in the propensity score model. These covariates could be considered composite variables. However, with multiple variables with varying specifications, results may differ.

### Future Research

This study does raise questions about the performance of propensity score models that are more complex, including more covariates and even interactions between covariates. More research on model-building strategies to construct the propensity score needs to be conducted. Of particular concern is the fact that iterative model-building algorithms (e.g., stepwise regression) are “designed to create good predictive models of [assignment]” (Brookhart et al., 2006, p. 1156). However, the goal of using the propensity score is not to predict assignment but to balance on the covariates, thus efficiently controlling confounding (Brookhart et al., 2006), and using iterative model-building algorithms may miss important confounders that are strongly related to outcome but weakly related to assignment (like LH). It also may reduce the overlap in propensity scores between the comparison and treatment groups.

Although there are varying recommendations for the number of strata that should be used, further research on how to determine the optimal number of strata to use to minimize bias is needed. In this study quintiles generally were more effective overall at adequately removing bias; however, the most effective number of strata varied depending on the covariate(s) included in the propensity score model. This leaves the question of how a researcher should determine the number of strata to use with rich data sets.

### Summary

Propensity score models have great potential to reduce bias in estimated effect sizes in quasi-experimental research. However, researchers must note that these models are not effective unless the propensity score model includes a covariate that is strongly related to *both* assignment and outcome. A pretest score often will meet this criteria. It is essential to include a “strong” covariate, but researchers also should include additional variables, which may further reduce bias, especially when using quintiles. The goal when creating propensity score models should be to create as rich a model as possible while including a strong covariate. Additionally, researchers must be aware that with larger number of strata, including two variables highly related to assignment but not including a variable highly related to outcome may not adequately reduce bias, potentially because those models result in only about 80% overlap of the treatment and comparison groups in propensity scores.

## Author Contributions

JLA came up with the initial idea and was in charge of drafting the Monte Carlo simulation code and the manuscript. JLA and DM developed the design of the work and interpreted the results. HR and JAA provided critical assistance to developing the code and conducting the analyses. TS made critical revisions of the manuscript for important intellectual content. All five authors (JLA, DM, HR, JAA, and TS) provided the final approval of the version to be published and agreed to be accountable for all aspects of the work in ensuring that questions related to the accuracy or integrity of any part of the work are appropriately investigated and resolved.

## Conflict of Interest Statement

JAA is owner of Adelson & Co., and TS is employed by LEAP. Neither of these authors were paid for their work on the manuscript and did not do the work as part of their workday at these commercial companies. Moreover, their companies do not benefit from this article. Therefore, we do not perceive a conflict of interest. The other authors declare that the research was conducted in the absence of any commercial or financial relationships that could be construed as a potential conflict of interest.
